# The potential of endoscopic ultrasound sonography (EUS)-elastography in determining the stage of pancreatic tumor 

**Published:** 2021

**Authors:** Afsaneh Saffarian, Pegah Eslami, Arash Dooghaie Moghadam, Faezeh Almasi, Mohamad Amin Pourhoseingholi, Hamid Asadzadeh Aghdaei, Amir Sadeghi, Mohammad Reza Zali

**Affiliations:** 1 *Gastroenterology and Liver Diseases Research Center, Research Institute for Gastroenterology and Liver Diseases, Shahid Beheshti University of Medical Sciences, Tehran, Iran. *; 2 *Department of Internal Medicine, Loghman Hakim Hospital, Shaheed Beheshti University of Medical Sciences, Tehran, Iran*; 3 *Department of Molecular Biology, Basic and Molecular Epidemiology of Gastrointestinal Disorders Research Center, Research Institute for Gastroenterology and Liver Diseases, Shahid Beheshti University of Medical Sciences, Tehran, Iran*

**Keywords:** Pancreatic neoplasms, Elasticity imaging techniques, Neoplasm staging

## Abstract

**Aim:**

The current study was designed to evaluate the role of semi-quantitative EUS- elastography (strain ratio) in staging malignant pancreatic lesions.

**Background:**

Pancreatic cancer is considered one of the most lethal malignancies with a survival rate of only 5% worldwide. Pancreatic lesions include a wide range of diagnoses from benign to malignant forms. Biopsy and pathological study are the gold standard for the differentiation of malignant lesions and staging of tumors. Recently, endoscopic ultrasound sonography (EUS) elastography has been noticed as a non-invasive diagnosis modality. Nevertheless, no evidence of its potential to determine different stages of malignant tumors is available.

**Methods:**

This prospective study included 81 adult patients with a confirmed diagnosis of malignant pancreatic lesion in different clarified stages. All diagnoses were confirmed after endoscopic ultrasound sonography via pathological investigation of surgical specimens or needle biopsies. The results of EUS-elastography based on tumor size (T staging), involved lymph nodes (N staging), and metastasis (M staging) were compared with the gold standard.

**Results:**

The mean age of patients was 60.11±13.57 years. The mean SR elastography value was 52.78±48.97. Elastography could not significantly discriminate T stage, N stage, or M stage of tumors (*p*=0.57, *p*=0.92, *p*=0.11, respectively). Moreover, the Spearman rank correlation coefficients for the correlation between T staging, N staging, M staging and SR elastography were not significant (*p*=0.40, *p*=0.94, *p*=0.39, respectively).

**Conclusion:**

The non-invasive modality EUS-elastography cannot replace the gold standard in staging tumors; however, EUS-elastography seemed to differentiate benign lesions from malignant ones.

## Introduction

 Presurgical diagnosis of solid pancreatic lesions (SPLs) remains a major clinical challenge and controversial subject for gastroenterologists. With a survival rate of less than 5% for 5 years, malignant pancreatic tumors make the most important group of SPLs (1). Pancreatic malignancy, known as intractable cancer, makes up 4.5% of all cancer-related deaths worldwide; however, according to GLOBOCAN (2018), it is considered the 11th most frequent malignancy in the world (2, 3). As the most common pancreatic lesion, adenocarcinoma is responsible for 70% to 95% of all solid malignancies (4). Currently, the only potential treatment for pancreatic cancer is surgery. At the time of presentation, around 15% of pancreatic tumors are resectable (5). Therefore, fast diagnosis and treatment initiation play critical roles in improving the survival rate of this malignancy (5). Today, endoscopic ultrasound-oriented fine needle aspiration (EUS-FNA) with sensitivity and specificity levels > 90% is the best diagnostic method for approaching pancreatic masses. Nevertheless, invasive approaches, as well as laparoscopic biopsy on occasions, are functional in individual cases (6). Moreover, as tumor size is an essential predictive factor in pancreatic cancer survival, contrast-enhanced multidetector computed tomography (CT) is typically recommended in diagnosing (7). After evaluating all the data, TNM staging can be calculated for most cases. This classification is normally used to characterize systematic and local pancreatic cancer and make the final decision for achieving the best survival rate and treatment approaches (8).

Since 2006, EUS elastography has been commonly used to approach solid pancreatic masses. This new ultrasound technique enables measuring the firmness of the target lesion non-invasively (9, 10). EUS elastography describes the stiffness level of a lesion in comparison to other densities as a qualitative and quantitative score (strain ratio; SR). In a meta-analysis, Mei et al. showed that EUS elastography is an efficient method for determining SPL type with sensitivity and specificity levels of 95% and 67%, respectively (11). Regarding the importance of fast decision-making about pancreatic masses, it was hypothesized that EUS elastography can be useful in determining the clinical staging of the tumor. This hypothesis was made based on previous studies that have demonstrated the relationship between elastography finding and invasive ductal carcinoma staging in breast cancer (12, 13).

This prospective study was conducted to evaluate the benefit of using EUS elastography to non-invasively assess pancreatic tumor staging. As the next step, the relationship between tumor size and elastography finding was also considered to determine the accuracy of this technique in the improvement of tumor classification. 

## Methods

**Patients**

This prospective single-center study was organized in Taleghani Hospital (affiliated with Shahid Beheshti University of Medical Sciences), a referral center for pancreatic disorders in Tehran, Iran. The population of this study included 81 patients who were diagnosed with malignant pancreatic tumors and underwent EUS-elastography in our center between October 2017 and 0ctober 2019. Patients younger than 18 years of age, had other types of pancreatic lesions, and those with liquid and cystic components were excluded from the study. CT-SCAN, EUS FNA biopsy, surgical specimen, and pathological findings were used to diagnose pancreatic cancer. 

Written informed constant was obtained from all participating patients. This investigation was approved by the Ethics Committee of Shahid Beheshti University of Medical Sciences. The experiments were conducted according to the World Medical Association’s Declaration of Helsinki for human-involved studies.

**EUS procedure and methods**

The entire EUS procedure was performed by an expert endosonographist using Olympus EUME2 (Olympus Corporation, Tokyo, Japan) coupled with an electronic ultrasound probe Olympus GF-UE 180. To perform the FNA biopsy, a Cook needle 22G was applied during the EUS procedure (Echotip; Wilson-Cook, Winston Salem, NC). Pancreatic masses were assessed using elastography. All endosonographic images and elastography videos were recorded and evaluated by an endosonographist who was blind to the final pathology diagnosis but informed about clinical and EUS findings. Moreover, to enhance the accuracy of the investigation, all videos were rechecked by a second expert endosonographist who had no previous knowledge about either pathology findings or clinical and EUS findings. A quantitative score, which is indeed a semi-quantitative elastography score, was used to show strain ratio. In this method, two endosonographic fields were chosen. The strain ratio was determined by dividing the normal region into the region of interest. The strain ratio means were determined and utilized as the ultimate result of each patient.

Patients were diagnosed according to a surgical specimen histology core needle biopsy or EUS-oriented fine needle aspiration (EUS-FNA). Contrast-improved multidetector computed tomography (CT) was performed, and the TMN staging score was recorded for all patients. Finally, the elastography findings, including quantitative scores, were compared with malignant mass sizes and cancer staging. For cancer staging, TMN staging was used and this score was calculated based on surgical, pathology, EUS, and CT-scan findings.

**Statistical Analysis**

EUS-elastography variables including tumor size, involved lymph nodes, and metastasis were described as frequency rates, percentages, and means and standard deviations. Tumor size means were compared using analysis of variance (ANOVA). Involved lymph nodes and metastasis were analyzed using an independent sample t-test. The correlation coefficients between factors were obtained by Spearman correlation test. A *p*-value of less than 0.05 (typically ≤ 0.05) was considered statistically significant. Statistical analyses were conducted using R software version 3.6.3.

## Results

In this prospective, single-center study, approximately 81 patients whose pathological tumor stages were determined with surgical methods and core needle biopsies were simultaneously evaluated with EUS-elastography. Then, the potential of EUS-elastography usage was determined by comparing its results with pathological gold-standard tests. All patients were investigated with EUS-EG, and no side effects or complications occurred. Only patients with a malignant tumor diagnosis were included in the current study; those with benign masses were excluded. After exclusion of those not meeting inclusion criteria, 81 eligible participants were enrolled in the study. The study population had a mean age of 60.11±13.57 years, ranging 19-89 years, with 56% of participants being male. The elastography values ranged 7.50-222 and had a mean of 52.78±48.97. With regard to staging based on tumor size (T staging), most patients were observed to be in the T4 stage (60.5%), and only 3.7% of the participants were categorized in the T1 stage (Table 1.). According to the current results, elastography could not significantly discriminate the T stage of the tumor or tumor size (*p*=0.57) (Table 2.) Furthermore, the Spearman’s rank correlation coefficient was not significant between T staging and the elastography strain ratio (*p*=0.40) (Table 3). The findings regarding number and location of the involved lymph nodes showed that 77.8% of participants belonged to the N0 stage (Table 1). Moreover, elastography could not significantly recognize the N stage of patients (*p*=0.92), nor did it correlate with the elastography ratio (*p=0*.94) (Table 2; Table 3). M staging revealed metastasis to other organs. Most patients were diagnosed with M0 stage (75.3%), and 24.7% of them were staged as M1 (Table 1). However, elastography was not effective in recognizing these three groups (*p*=0.11) (Table 2). Nevertheless, no correlation was detected between M staging and the elastography strain ratio (*p*=0.39) (Table 3).

**Table 1:** Prevalence and frequency of different stages of T staging, N staging, M staging of patients with malignant pancreatic lesions

Valid		Frequency	Prevalence (%)
T staging*	T1 T2 T3 T4	3 24 5 49	3.7 29.6 6.2 60.5
N staging*	N0 N1	63 18	77.8 22.2
M staging*	M0 M1	61 20	75.3 24.7

*T staging: tumor size staging; N staging: involved lymph nodes; M staging: metastasis staging.

**Table 2:** T test results of comparison of elastography ratios in differentiation of different stages of malignant pancreatic tumors

valid		Elastography strain ratio	*P* value
T staging*	T1 T2 T3 T4	25.25±25.10 42.78±31.05 63.87±56.91 57.92±55.83	0.57
N staging*	N0 N1	52.42±48.33 53.93±52.73	0.92
M staging*	M0 M1	48.86±46.95 72.64±55.75	0.11

*T staging: tumor size staging; N staging: involved lymph nodes; M staging: metastasis staging.

**Table 3:** Spearman’s rank correlation coefficients of T staging, N staging, M staging, and elastography strain ratio

Indexes	Elastography strain ratio	
Valid	r	*p* value
T staging*	0.107	0.401
N staging*	0.009	0.944
M staging*	0.107	0.399

*T staging: tumor size staging; N staging: involved lymph nodes; M staging: metastasis staging

## Discussion

This prospective study was designed to evaluate the proficiency of elastography in discriminating different stages of solid pancreatic tumors. Malignant pancreatic lesions were studied according to stiffness score (strain ratio). It was observed that elastography could not be considered an effective modality for distinguishing between pancreatic tumor stages. Pancreatic lesions are usually detected incidentally or in routine radiographic evaluations, including ultrasound or CT-scan. The findings of these modalities include pancreatic masses, tumors, cysts, or benign nodules. Although most of these lesions are benign, their malignant forms should be surgically resected. Therefore, pathological investigations are vital in pancreatic lesions (14). Several methods were considered to determine focal lesions. However, their effectiveness is unknown in many situations (15). In EUS, an endoscope with a camera enters the body cavity and maps the tissue characteristics via ultrasound waves. Recent studies have shown that images obtained from EUS could more efficiently detect pancreatic lesions compared with previous methods such as US or CT-scan. However, this technique has only 60% accuracy (15). Pancreatic lesions are hypoechoic and irregular in EUS; therefore, it is difficult to differentiate between malignant and benign lesion types via EUS (15). Thus, a new version of EUS has been introduced: EUS elastography, in which not only are EUS facilities utilized through elastography, but lesion stiffness can also be determined. This modality was designed based on the fact that malignancy can change tissue hardness. Elastography creates compression with ultrasound and then checks any small shift/movement in the tissue (16). Recently, the potency of this technique has been considered in differentiating malignant lesions from benign ones, especially in pancreatic lesions (15). A recent European study with 258 participants reported the specificity of this modality in recognizing malignant tumors from others as 66%, which appears to be more than EUS specificity (17). However, this modality could not replace biopsy study via EUS-FNA (17). Contrary to previous studies, Itoh et al. (18) reported the optimal cut-off of 57.4% as the severe mark of pancreatic fibrosis. They believed that EUS-EG was significantly more potent than previous modalities. They estimated the diagnosis specificity and accuracy to be 91.8% and 89.7%, respectively. Nevertheless, one of the limitations of their study in comparison with previous studies was the small population. Itoh et al. (2015) (19) evaluated the role of magnetic resonance elastography in detecting cancerous lesions from normal pancreatic tissues. They revealed that MRE could significantly distinguish between malignant and normal tissues (19). Another similar study was carried out by Itokawa et al. (2011) (20) based on the strain ratio determining the hardness of pancreatic masses. They showed that although this method had good sensitivity in diagnosing malignant tumors, it could not distinguish between malignant masses and benign forms with acceptable specificity. In line with their findings, the current results showed that this modality could not distinguish between different stages of malignancy based on stiffness. However, this study did not evaluate the role of this modality in recognizing malignant masses from benign forms (20). The mean strain ratio was 52.78 among the group of patients with malignant tumors in different stages. This is similar to cut-offs in other studies comparing the stiffness of pancreatic tumors with the normal tissue of the pancreas. In their prospective study, Dawwas et al. (21) simultaneously measured the synergic effects of the strain ratio and mass elasticity using means of EUS-EG. They concluded that although the measurements could detect malignant masses with high sensitivity, they did not have optimal accuracy or specificity (21). Thus, it appears that this modality cannot be an alternative option for biopsy sampling, although it contributes to EUS-FNA (21). Furthermore, they estimated that SR above 59.25 strongly represented malignancy (21). This number is close to the SR mean reported in our study on malignant tumors. In the current study, the effectiveness of EUS-EG was evaluated in terms of tumor size staging (T), number of lymph nodes involved (N), and metastasis to other organs (M). The results revealed no significant effects of this method in distinguishing between different stages of pancreatic malignant tumors. Moreover, no correlation was observed between staging and the EUS-EG strain ratio. However, Spearman's rank correlation coefficient showed that with increasing stiffness, stages also increased. According to the results, by accepting the effectiveness of quantitative SR via EUS-elastography, this modality could not significantly differentiate between various stages of pancreatic tumors. Therefore, it cannot help sufficiently determine the treatment strategy, and biopsies are constantly needed. However, further investigation with a larger study population is needed to evaluate the current results. 

This study had several limitations, including a small population size, a single setting, and no comparison between different types of pancreatic lesions, including benign or malignant. Thus, we believe that this study could be used as a cornerstone for future analyses. 

This study investigated the role of EUS-elastography as a non-invasive diagnosis modality in distinguishing between different stages of pancreatic tumors in terms of tumor size, number of involved lymph nodes, and far metastasis. However, it appears that the studied modality had no significant effects in differentiating tumor stages in malignant lesions. Furthermore, the staging was not correlated with the SR of elastography.

## Conflict of interests

The authors declare that they have no conflict of interest.
